# Production of chickens with green fluorescent protein-knockin in the Z chromosome and detection of green fluorescent protein-positive chicks in the embryonic stage

**DOI:** 10.5713/ab.22.0405

**Published:** 2023-02-26

**Authors:** Kyung Soo Kang, Seung Pyo Shin, In Su Ha, Si Eun Kim, Ki Hyun Kim, Hyeong Ju Ryu, Tae Sub Park

**Affiliations:** 1 Depatment of Bio Life Science, ShinGu University, Seongnam 13174, Korea; 2 Institute of Green-Bio Science and Technology, Seoul National University, Pyeongchang 25354, Korea; 3 Graduate School of International Agricultural Technology, Seoul National University, Pyeongchang 25354, Korea

**Keywords:** Chicken, CRISPR, Cas9 Nuclease, Embryo Sexing, Knockin, Primordial Germ Cell

## Abstract

**Objective:**

The clustered regularly interspaced short palindromic repeat (CRISPR)/CRISPR-associated protein 9 (Cas9) system, which is the most efficient and reliable tool for precisely targeted modification of the genome of living cells, has generated considerable excitement for industrial applications as well as scientific research. In this study, we developed a gene-editing and detection system for chick embryo sexing during the embryonic stage.

**Methods:**

By combining the CRISPR/Cas9 technical platform and germ cell-mediated germline transmission, we not only generated Z chromosome-targeted knockin chickens but also developed a detection system for fluorescence-positive male chicks in the embryonic stage.

**Results:**

We targeted a green fluorescent protein (*GFP*) transgene into a specific locus on the Z chromosome of chicken primordial germ cells (PGCs), resulting in the production of *Z^GFP^*-knockin chickens. By mating *Z^GFP^*-knockin females (*Z^GFP^*/W) with wild males (Z/Z) and using a *GFP* detection system, we could identify chick sex, as the *GFP* transgene was expressed on the Z chromosome only in male offspring (*Z^GFP^*/Z) even before hatching.

**Conclusion:**

Our results demonstrate that the CRISPR/Cas9 technical platform with chicken PGCs facilitates the production of specific genome-edited chickens for basic research as well as practical applications.

## INTRODUCTION

Avian species have a distinguished history as a model system in developmental and biological research [1]. To modify the specific genomic locus of living cells, the clustered regularly interspaced short palindromic repeat (CRISPR)/CRISPR-associated protein 9 (Cas9) system would be the most efficient, easiest, and most precise tool [2,3]. The state-of-the-art CRISPR/Cas9 system has revolutionized bioscience and biotechnology because of its simplicity and high-efficiency editing of the genomes of various species [4–7]. Using the SCNT cloning technique and direct injection into one-cell-stage embryos, CRISPR/Cas9-mediated knockout (KO) and knockin (KI) animals have been successfully generated, particularly in mammals [5–7]. However, it is too difficult to generate gene-knockin or gene-knockout avian species using the CRIPSR-Cas9 technical platform because of practical difficulties associated with the development of these techniques in mammals [8–11].

Recently, germline-transmittable primordial germ cells (PGCs) were established and used as an alternative strategy for the production of genetically altered chickens [8–11]. Using CRISPR/Cas9 technical platforms, production of specific genome-tailored KO and KI chickens will make important contributions to advancements in bioscience via their use as avian models and through embryonic development studies. In addition, the potential of genome-edited chickens mediated by the CRISPR/Cas9 system reaches beyond basic research; locus-specific genome engineering will be widely used in agriculture and industry because of the lack of exogenous transgene integration.

To produce KO chickens, Park et al [10] adapted the CRISPR-Cas9 system to chicken PGCs and generated G0/G1 switch gene 2 (*G0S2*)-mutated chickens. G0S2 inhibits adipose triglyceride lipase (ATGL), which is a lipolysis catalase in adipose tissue. Thus, G0S2 KO chickens have dramatically reduced lipid content and fat deposition [10]. Genetic improvement of economically important traits such as a muscle growth is a major research issue in the livestock industry, and myostatin (*MSTN*) would be a strong candidate gene for genetic modification because of the double-muscling growth in MSTN mutant animals [11]. Kim et al [11] mutated the *MSTN* gene in chickens using D10A-Cas9 mutant nickase, and MSTN KO chickens showed a significant increase in skeletal muscles. Subsequently, these gene-edited chickens could be used to investigate regulatory mechanisms and interactions in metabolic pathways as an animal model, as well as in industrial applications.

Lee et al [12] targeted the green fluorescent protein (*GFP*) transgene specifically into the Z chromosome in chickens to develop an avian sexing model. The *GFP* transgene was robustly expressed in Z chromosome KI chickens, and this expression could be used for sex identification during embryogenesis [12]. However, there is no detection system for GFP expression during embryonic developmental stages in Z chromosome KI chickens. In this study, we generated Z chromosome-targeted KI chickens and developed a detection system for fluorescence-positive male chicks in the embryonic stage.

## MATERIALS AND METHODS

### Experimental animal care

The procedures followed in the care of chickens for experimental use in this study were approved by the Institutional Animal Care and Use Committee (SNU-150825-2-1), Seoul National University. The procedures used in the care of chickens for experimental use were approved by the Institute of Laboratory Animal Resources, Seoul National University. Chickens were maintained according to a standard management program at the University Animal Farm, Pyeongchang campus, Seoul National University, Korea. The procedures for animal management, reproduction, and embryo manipulation adhered to the standard operating protocols of our laboratory.

### Chicken PGC culture and FACS sorting after transfection

Chicken PGCs from White Leghorn or a commercial line (Hy-Line Brown) were maintained and subpassaged in knockout Dulbecco’s modified eagle’s medium (Invitrogen, Waltham, MA, USA) supplemented with 20% fetal bovine serum (Invitrogen, USA), 2% chicken serum (Sigma-Aldrich, St. Louis, MO, USA), 1× nucleosides (Millipore, Temecula, CA, USA), 2 mM L-glutamine, 1× nonessential amino acids, β-mercaptoethanol, 10 mM sodium pyruvate, 1× antibiotic–antimycotic (Invitrogen, USA), and human basic fibroblast growth factor (10 ng/mL; Koma Biotech, Seoul, Korea). Chicken PGCs were cultured in an incubator at 37°C with an atmosphere of 5% CO_2_ and 60% to 70% relative humidity. The cultured PGCs were subcultured onto mitomycin-inactivated mouse embryonic fibroblasts (MEFs) at 5 to 6 d intervals by gentle pipetting without any enzymatic treatment. For the Z chromosome KI experiment, 7.5 μL Lipofectamine 3000 Reagent were diluted in 250 μL OPTI-MEM (Invitrogen, USA), and the Cas9 expression plasmid, Z chr gRNA, and donor GFP KI vector (1.5:1:3.0 μg) were mixed with Lipofectamine P3000 Reagent in 250 μL OPTI-MEM at room temperature. After incubation for 5 min, the two mixtures were combined and incubated for an additional 20 min. The mixture was gently pipetted and dropped onto the cultured chicken PGCs. After incubation at 37°C in 5% CO_2_ for 4 h, PGCs were gently washed with phosphate buffered saline (PBS) three times, and fresh culture medium was added. One day after lipofection, GFP-expressing cells were sorted using a FACSAria III cell sorter (Becton, Dickinson and Company, Franklin Lakes, NJ, USA). After harvesting, the chicken PGCs were resuspended in PBS containing 0.1% BSA and strained through a cell strainer for FACS separation (40 μm; BD Falcon; Becton, Dickinson and Company, USA). After sorting, cells were regrown on a mitomycin-inactivated MEF feeder.

### Information regarding the targeted locus and genomic polymerase chain reaction

Genomic polymerase chain reaction (PCR) was performed using an initial incubation at 94°C for 5 min, followed by cycles of denaturation, annealing, and extension for each target gene or locus using the corresponding specific primer sets (Table 1). The reaction was terminated by a final incubation at 72°C for 7 min. Sequence information on the chicken targeted sequences on Z chromosome (25,814,118-25,814,865 on Z chr.) were downloaded from the UCSC Genome Browser (http://genome.ucsc.edu).

### Transplantation of chicken PGCs and testcross analysis for screening of donor germ cell-derived KI chicks

To transplant chicken Z chr GFP KI PGCs into recipient embryos, a small window was made on the pointed end of the recipient eggs, and a 2 μL aliquot containing >3,000 PGCs was microinjected with a micropipette into the dorsal aorta of the recipient embryo. The egg window of the recipient embryo was sealed with paraffin film, and the egg was incubated with the pointed end down until hatching. After sexual maturation, only male chicks were used for testcross analysis because the Z chr GFP KI PGCs were established from a male embryo. For the generation of Z chr KI chickens, WL was used as the recipient embryos; the putative germline chimeras were also mated with WL hens because the Z chr GFP KI germ cell-derived chicks could be identified by GFP expression. The hatched green transgenic offspring could be screened out using a fluorescent excitation lamp with a detection filter (BLS Ltd., Budapest, Hungary).

### System for detecting GFP-expressing chicks

The fertilized eggs of *Z^GFP^*-knockin females (*Z^GFP^*/W) mated with wild males (Z/Z) were incubated at 37.5°C and 70% relative humidity while being rocked at a 90-degree angle at 60 min intervals. For detection of GFP-expressing chick embryos, circle windowing was carefully performed at the blunt end with a modified medical drill (approximately 8 mm in diameter holed drill) at 10 to 12 days of incubation. Subsequently, the inner and outer shell membranes of the egg air sac were removed. GFP expression was visualized through the whole egg by illuminating the chick embryo with light of a peak intensity at the emission wavelength of 488 nm (blue LED laser; Beijing Toplaser Technology Co. Ltd., Beijing, China), and the excitation signal was detected with a narrow-range filter for the wavelength 510 nm (Huidongbao Technology Co. Ltd., Shenzhen, Guangdong, China).

## RESULTS AND DISCUSSION

### Production of *Z^GFP^*-knockin primordial germ cells

**Figure 1:**
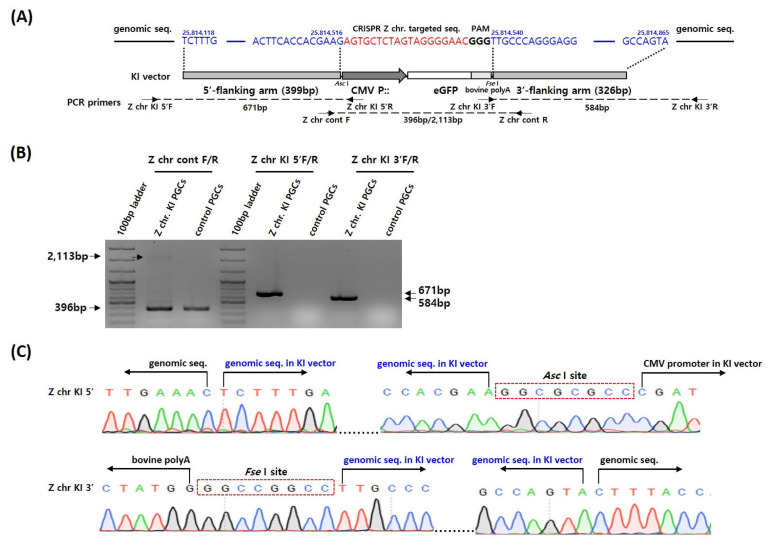
Schematic designs of the CRISPR/Cas9-mediated knockin (KI) on the Z chromosome and insertion analysis in chicken primordial germ cells (PGCs). (A) Sequences of the target sites of CRISPR Z chr gRNA, and KI vector design. Sequences in red indicate the target sites of CRISPR Z chr gRNA, and the bold ‘GGG’ sequences are the protospacer adjacent motif (PAM) sequences. Sequences in blue indicate the 5′- and 3′-flanking arm positions on the Z chromosome. Arrows indicate the positions of the polymerase chain reaction primer sets. (B) PCR analysis of Z chr KI PGCs using the appropriate primer sets. The Z chr cont F/R primer set detected both wild and donor KI GFP vector-inserted loci with different PCR product sizes (396 vs 2,113 bp). The Z chr KI 5′F/R and Z chr KI 3′F/R primer sets amplified only the targeted loci. (C) Chromatogram sequences of the CRISPR Z chr target site. Both the 5′- and 3′-flanking regions of the KI vector containing the CMV-GFP transgene were precisely integrated into the intended locus with no nucleotide deletion or addition.

We designed a gRNA targeting the intergenic region between doublesex and mab-3 related transcription factor1 (*DMRT1*) and *DMRT3* on the chicken Z chromosome (Figure 1A) and constructed a donor KI plasmid harboring the GFP expression transgene with 399 bp 5′- and 326 bp 3′-flanking genomic sequences (Figure 1A). The Z chr gRNA and donor KI plasmids were applied to chicken PGCs, and insertion of the CRISPR/Cas9-mediated donor KI plasmid into the target site was confirmed by PCR analysis. The donor KI plasmid was integrated into the targeted locus on the Z chromosome (Figure 1B). In addition, sequencing analysis of the 5′- and 3′-flanking regions of the inserted locus revealed that the sequences were targeted precisely, with no nucleotide deletion or insertion in the genomic or donor KI plasmid sequences (Figure 1C).

### Generation of *Z^GFP^*-knockin GFP-expressing chicks through testcross analysis

**Figure 2:**
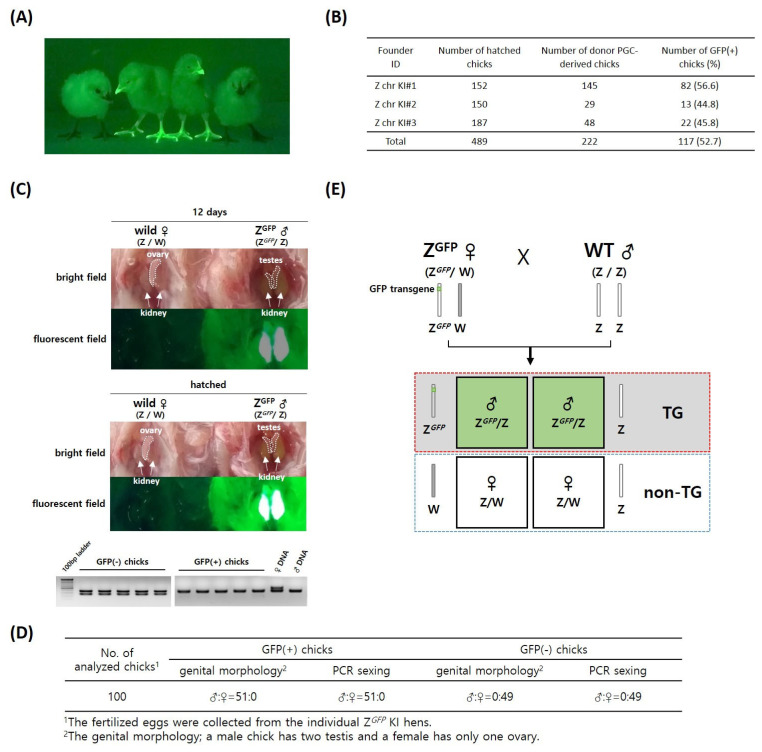
Production and detection of green fluorescent protein (GFP)-expressing offspring from the germline chimeras transplanted into Z chr GFP KI PGCs. (A) GFP-expressing offspring from testcross analysis. (B) Production efficiencies of GFP-expressing offspring from the germline chimeras. (C) Detection of GFP expression in male chicks only. Following mating of *Z^GFP^* KI females (*Z^GFP^*/W) and wild males (Z/Z), male offspring (*Z^GFP^*/Z) were identified by Z chr-GFP transgene expression during the embryo stage and after hatching. Chick sex was confirmed by genital morphology (upper panel) and genomic polymerase chain reaction (lower panel). (D) Comparison of hatched chick sex determination by GFP transgene expression, genital morphology, and genomic PCR sexing. (E) Predetermination model of chick sexing. Before hatching, male chicks can be screened out by detecting expression of the *GFP* transgene inherited from hens with *Z^GFP^*/W. Female chicks do not contain the exogenous transgene.

Subsequently, we generated Z chr-KI GFP-expressing chicks from germline chimeras through testcross analysis (Figure 2A and 2B). The production efficiencies of GFP-positive chicks from the germline chimeras ranged from 44.8% to 56.6% (mean, 52.7%) (Figure 2B). To confirm the Z chr-targeted KI in GFP-positive offspring, genomic PCR analysis was conducted; all GFP-expressing progeny were identified as Z chr-targeted chicks (Supplementary Figure S1A). The CRISPR/Cas9-mediated knockin (KI) target genomic position on the Z chromosome was cloned and sequenced in Z chr-KI GFP-expressing chicks. The information on the targeted genomic site and KI vector was confirmed in Z chr-KI chicks (Supplementary Figure S1B). The KI target site was precisely positioned between the *DMRT1* and *DMRT3* genes on the Z chromosome (Supplementary Figure S1C).

After sexual maturation of the Z chr-KI GFP-expressing chickens, we mated *Z^GFP^* KI females (*Z^GFP^*/W) and wild males (Z/Z). Only male offspring exhibited Z chr-GFP transgene expression (*Z^GFP^*/Z), even before hatching (Figure 2C). To evaluate the accuracy of GFP expression-dependent sex determination, many GFP-positive and GFP-negative progeny from nine hens were screened by assessing genital morphology and PCR sexing before and after hatching (Figure 2C and 2D). The results of chick sex determination by GFP expression targeted to the Z chromosome were identical to those of genomic PCR and genital morphology (Figure 2C–2E).

### Screening system for detecting GFP-expressing chicks

**Figure 3:**
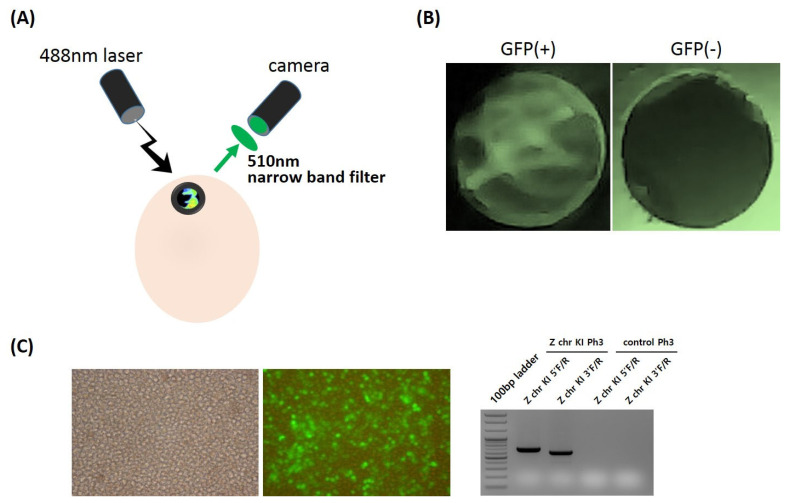
*In ovo* sexing screening system for green fluorescent protein (GFP)-expressing embryos at early developmental stages. (A) The pre-hatch sex determination system of GFP-expressing embryos using a UV light excitation device (488 nm) and an emission filter (510 nm). (B) Detection of GFP visualization from Z chr-targeted transgenic embryos (left panel: GFP-expressing embryo, right panel: control embryo).

In the next experiment, we developed an *in ovo* sexing screening system for detection of GFP-expressing embryos in the early developmental stages using a UV light excitation device and emission filters for GFP visualization from Z chr-targeted transgenic embryos (Figure 3A). For expression of GFP inserted into the Z chromosome, we used cytomegalovirus (CMV) promoter, which is strongly and ubiquitously expressed in most chicken tissues. Thus, Z chr-targeted KI male offspring expressed the GFP transgene in the majority of their tissues and could be detected with an *in ovo* sexing image screening system of UV excitation and emission filters (Figure 3B). By contrast, non-transgenic female chicks as a control did not show any GFP-positive signal (Figure 3B). For industrial application, we isolated and cultured primordial germ cells from a commercial layer line, Hy-Line Brown, and applied the Z chr-targeted platform to the PGC line (Figure 3C). We successfully integrated the CRISPR/Cas9-mediated Z chr-targeted KI system into a commercial layer line and confirmed it with genomic PCR analysis (Figure 3C).

Currently, one of the biggest challenges facing the chicken egg industry is culling male chicks post-hatching. Male chicks in the egg laying industry are culled shortly after hatching because they cannot produce eggs and are not suitable for meat production. Pre-hatch sex determination is needed not only to eliminate ethical issues but also to improve animal production efficiency and reduce costs. Thus, the development of *in ovo* sexing systems for animal welfare should be necessary for industrial application. Based on CRISPR/Cas9-mediated chicken genome engineering, the sex selection process at embryonic stages could solve the ethical and welfare concerns and be applied for sustainability of the egg laying industry in the future. However, the public acceptance and regulation for human consumption of genetically modified livestock should be further discussed although the layer hens do not contain any transgene after in ovo sexing.

Although KO chickens have been produced by conventional homologous recombination gene targeting and transcription activator-like effector nuclease (TALEN) techniques [9,13], the versatile CRISPR/Cas9 system is more efficient, particularly for large-scale and high-throughput precise genetic changes in avian species, and is not time consuming or complex. Because of increasing interest from the agriculture industry, practical methods of altering the poultry genome have been sought for the last three decades. The data from our study shown in Figure 2E suggest that predetermination of chick embryo sex before hatching could be useful for the poultry industry because of the lack of exogenous transgene integration. In addition, the chicken has been used as a model species in research and continues to serve as an important experimental model in developmental biology and pathology. Future advancements in and use of genetically modified chickens as model animals will be facilitated by the state-of-the-art CRISPR/Cas9 technical platform.

## Figures and Tables

**Table 1:** Sequences of primers used for genomic PCR amplification

Gene	Forward primer	Reverse primer	Annealing temp. (°C)	Product size (bp)
Z chr control	5′-AGCAAGATCAGGAAGGTGCC-3′	5′-TACTGGCAGCATGCACAAGA-3′	65	396 or 2,113
Z chr KI 5′	5′-CAGCGTCGTTTACATGCCTT-3′	5′-GGCTATGAACTAATGACCCCG-3′	65	671
Z chr KI 3′	5′-CCACTCCCACTGTCCTTTCC-3′	5′-AACACCAGGAAGCAACGGAT-3′	65	584
Sexing PCR	CPE15F 5′-AAGCATAGAAACAATGTGGGAC-3′ USP1 5′-CTATGCCTACCACMTTCCTATTTGC-3′	CPE15R 5′-AACTCTGTCTGGAAGGACTT-3′ USP3 5′-AGCTGGAYTTCAGWSCATCTTCT-3′	60	252 374

PCR, polymerase chain reaction.

## SUPPLEMENTARY MATERIAL

**Supplementary Figure S1**. (A) PCR analysis of Z chr KI offspring using the Z chr KI 5′F/R and Z chr KI 3′F/R primer sets. (B) CRISPR knockin (KI) target position on the Z chromosome and sequence information of the targeted genomic site and KI vector. Red underlined sequences denote PCR primer sets. (C) The position of the target site between the DMRT1 and DMRT3 genes on the Z chromosome.
