# Supplementary material for: Production of chickens with green fluorescent protein-knockin in the Z chromosome and detection of green fluorescent protein-positive chicks in the embryonic stage
Source: Anim Biosci. 2023 Feb 26;36(6):973–9. doi: 10.5713/ab.22.0405 (PMC10164537; doi:10.5713/ab.22.0405)
Supplement: Supplementary file 1 [file ab-22-0405-Supplementary-Fig-1.pdf]

Suppl Figure.

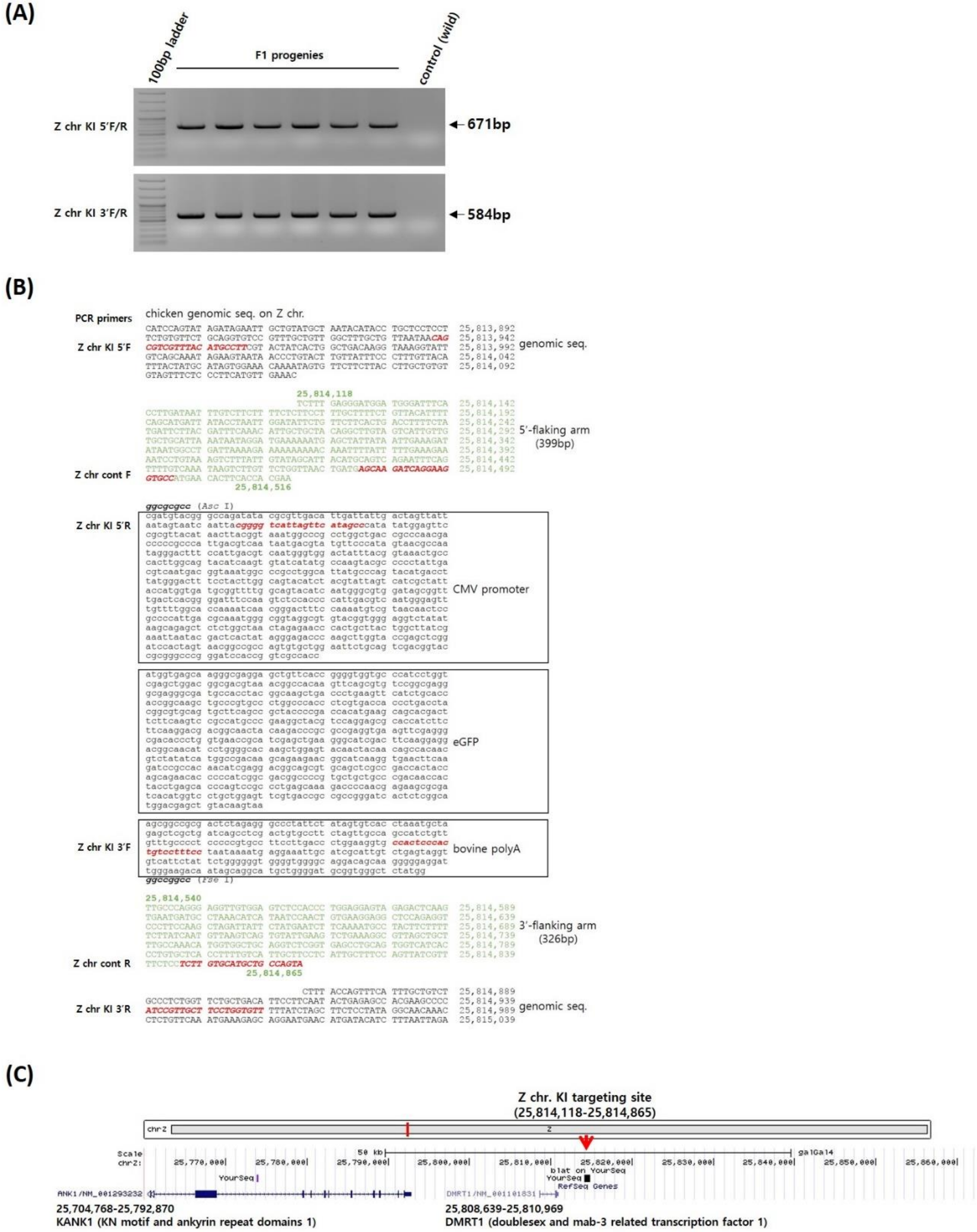

Supplementary Figure S1. (A) PCR analysis of Z chr KI offspring using the Z chr KI 5'F/R and Z chr KI 3'F/R primer sets. **(B)** CRISPR knockin (KI) target position on the Z chromosome and sequence information of the targeted genomic site and KI vector. Red underlined sequences denote PCR primer sets. **(C)** The position of the target site between the *DMRT1* and *DMRT3* genes on the Z chromosome.
